# Treating Unmet Needs in Psychiatry (TUNE-UP): targeted service increases out-patient initiations of clozapine

**DOI:** 10.1192/bjo.2026.10988

**Published:** 2026-02-26

**Authors:** Zmarak Ahmad Khan, Ioana Varvari, Valentina Mancini, Chambrez Zita-Zauchenberger, Shashwati Kantor, Jack B. Fanshawe, Sharon Musiiwa, Alexandra Pledge, Benjamin Pearce, Saik J. G. N. de La Motte de Broöns de Vauvert, Digby Quested, Daniel Maughan, Joseph Baskerville-Butler, Philip McGuire, Oliver D. Howes, Toby Pillinger, Robert A. McCutcheon

**Affiliations:** Psychiatry, University of Oxford, UK; Oxford Health NHS Foundation Trust, Oxford, UK; TUNE-UP Service, Oxford Health NHS Foundation Trust, Oxford, UK; University of Oxford Wellcome Centre for Integrative Neuroimaging, UK; Warneford Hospital, Oxford Health NHS Foundation Trust, Oxford, UK; Psychosis Studies, South London and Maudsley Mental Health NHS Trust, London, UK; Department of Psychosis Studies, King’s College London Institute of Psychiatry, Psychology & Neuroscience, UK; TUNE-UP Service, South London and Maudsley Mental Health NHS Trust, London, UK

**Keywords:** Psychotic disorders/schizophrenia, community mental health teams, general adult psychiatry, antipsychotics, out-patient treatment

## Abstract

**Background:**

In the UK, clozapine is the only licensed treatment for treatment-resistant schizophrenia (TRS). However, it is underused because initiation is often limited by the need for in-patient admission, which is costly and unattractive to patients. Community clozapine services may address this.

**Aims:**

To describe a targeted out-patient clinic (Treating Unmet Needs in Psychiatry (TUNE-UP)) for TRS management and assess its impact on community clozapine initiation rates.

**Method:**

We reviewed clozapine titrations of patients under four community mental health teams from September 2021 to January 2025, recording whether titration occurred as in- or out-patient. The TUNE-UP clozapine clinic operated for 12 months (September 2023 to September 2024). Initiation rates during the TUNE-UP period were compared with those when the service was unavailable, using Poisson regression. Clinical outcomes were assessed using scales including the Positive and Negative Syndrome Scale (PANSS).

**Results:**

Fifty-one individuals commenced clozapine during the study period. There was a significant increase in the rates of community initiation in the TUNE-UP period (11.0 per year) compared with those outside of this period (1.71 per year; incidence risk ratio 6.42 [95% CI 2.04–20.2, *P* = 0.0015]). Patients seen by TUNE-UP showed significant improvements in PANSS (*n* = 6, median improvement 21.5 [95% CI 7.0–33.0], *P* = 0.03).

**Conclusions:**

A specialist service was associated with a significant increase in community clozapine initiations. This approach offers a strategy to improve TRS treatment in the community.

Schizophrenia is a psychiatric disorder consisting of positive, negative and cognitive symptoms, and is associated with significant functional impairment.^1^ Nearly one in four patients with first-episode psychosis or schizophrenia will develop treatment-resistant schizophrenia (TRS) in the early stages of treatment.^2^ TRS is defined as an inadequate response to treatment of adequate dose, duration and concordance with two different antipsychotics.^3,4^ National Institute for Health and Care Excellence (UK) guidelines advise that clozapine be considered after 2 antipsychotics (1 an atypical) have been tried for 6–8 weeks and an inadequate effect has been demonstrated.^4^

Clozapine is the only antipsychotic treatment that is specifically licensed in the UK for use in TRS, and it is associated with better outcomes compared with other antipsychotics in this patient population.^3–6^ Despite guidelines advising treatment with clozapine at the earliest opportunity in TRS, there can be significant delays to its initiation in clinical practice,^7–9^ and this is associated with worse outcomes.^10,11^

Delays in clozapine initiation can result, in part, from the fact that initiation is often available only in an in-patient setting. This is typically an unattractive option for patients and incurs significant financial cost.^12,13^ In the UK there had been a historical requirement under the product licence for clozapine to be started in hospital; although this was revoked in 2002, the majority of clozapine initiations have continued to take place in in-patient settings.^14^ Community titrations of clozapine have previously been shown to be a safe and feasible approach, and are associated with significant reductions in cost, service use and symptom severity.^12^ The impact of a specialist community initiation service on clozapine initiation rates in the community, however, is yet to be investigated.

This study aimed to investigate whether a community clozapine titration service is associated with an increase in the rate of community clozapine titrations. It was hypothesised that there would be an increase in community clozapine titrations over the period during which this service was available, compared with periods when it was not available. Comparison of key clinical outcomes at baseline and discharge, in patients titrated through this service, were analysed as secondary objectives.

## Method

A retrospective analysis of clozapine titration locations over 40 months (September 2021–January 2025) was conducted. This period comprised 2 years as a baseline prior to the service’s commencement, the 1 year when this service was operational, followed by the 4-month period immediately after its cessation. During 12 of these months (period 3) the specialist community service, the Treating Unmet Needs in Psychiatry (TUNE-UP) clinic, was in operation.

### Objectives

The primary objective was to compare the rates of community clozapine titration in the year in which the TUNE-UP service was running with the 2 years beforehand, and the 4-month period afterwards. A sensitivity analysis was also performed excluding the post-TUNE-UP period. Secondary objectives included further analyses of combined (i.e. community and in-patient) titration rates and of first-time and re-titrations analysed separately. An exploratory analysis of clozapine initiation on clinical measures in TUNE-UP patients was also performed, and data regarding the length of stay for in-patient clozapine initiations were obtained.

### Service description

The TUNE-UP clinic launched in September 2023 as a satellite service to four Oxford Health NHS Foundation Trust (UK) community psychiatry teams (three adult mental health teams (AMHTs) and one early intervention service). These teams serve a population of approximately 165 000 people and have a combined caseload of 1455 patients.

The TUNE-UP team consisted of one full-time medical doctor, with additional support provided by clinical academics and the Physical Health in Severe Mental Illness team.

Patients were referred by these teams to TUNE-UP for assessment and management advice for significant positive, negative and cognitive symptoms or physical health concerns. A comprehensive assessment was performed (see Box 1), and a management plan developed in collaboration with the patient. Following approval by the referrer, the TUNE-UP service then implemented the proposed plan, including, but not limited to, community initiation of clozapine (see Box 2, based on existing community clozapine initiation approaches).^15,16^ Following clozapine initiation, and once clinical stability was achieved, patients were discharged from TUNE-UP back to the sole care of their team.

Box 1 The Treating Unmet Needs in Psychiatry (TUNE-UP) clinic service model

From referral, within 3 weeks:

- Review patient electronic notes and obtain collateral information.
- Explore and help patients articulate and formulate their goals.

Conduct a comprehensive assessment including standardised rating scales.

- Positive and Negative Syndrome Scale;^17^
- Brief Negative Syndrome Scale;^18^
- Calgary Depression Scale;^19^
- Screen for Cognitive Impairment in Psychiatry;^20^
- Subjective Scale to Investigate Cognition in Schizophrenia;^21^
- Sheehan Disability Scale;^22^
- Social and Occupational Functioning Assessment Scale;^23^
- Sleep Condition Indicator.^24^

1. Investigate physical health via physical examination and clinical investigations, including electrocardiogram and blood tests (prolactin, full blood count, erythrocyte sedimentation rate, thyroid function tests, liver function tests, urea and electrolytes, bone profile, lipids profile, HbA1c, c-reactive protein, troponin-I (for clozapine titrations) and, if indicated, psychotropic levels).

From investigation stage to management formulation stage, 1 week:

- Identify treatment options for a range of symptom domains: positive symptoms, negative symptoms, affective symptoms, sleep, cardiometabolic health.
- Develop treatment strategies via a Co-Care Approach Model (involving adult mental health teams (AMHTs), general practitioners (GPs) and carers) aligned with patient goals, such as side-effect informed treatment optimisation,^25^ side-effects management and adjunctive therapies including psychosocial interventions.

Implementation (3–6 months)

- Implement and monitor changes.
- Longitudinal assessment with repetition of clinical scales at discharge.
- Discharge to AMHT or GP with short- and long-term recommendations that align with patient goals.

Box 2 Community clozapine initiation procedures

After a patient meets the treatment resistance criteria, the following steps are conducted:

- community initiation eligibility screen as per Maudsley Guidelines;^3^
- baseline investigations: electrocardiogram, full blood count (FBC), troponin-I, c-reactive protein (CRP), liver function tests, urea and electrolytes, glucose, lipids, HbA1c.

Considering patient preference, a tailored titration plan weighing mental state, current psychotropics and prior trials/responses to clozapine is designed. A standard plan is:

- Week 1: start at 6.25 mg and double dose every other day reaching 50 mg at the end of week 1.
- Weeks 2–5: increase dose by 25 mg/day.
- Week 5: consultant-guided increments of 25–50 mg/day until a stable dose is reached, aiming for therapeutic plasma levels (0.35–0.50 mg/L).

This titration rate follows other community titration protocols. Slower titration allows for detection of adverse effects earlier, improves tolerability and may reduce the risk of certain adverse effects that are titration rate-dependent.^3,15^

Monitoring includes:

- Physical health checks include pulse, postural blood pressure, temperature, respiratory rate, oxygen saturations.
  - weeks 1 and 2, 3 times per week;
  - weeks 3 and 4, 2 times per week;
  - weeks 5 and 6, 1 time per week.
- Haematological monitoring:
  - troponin-I and CRP weekly for 6 weeks;
  - FBC weekly until week 18, fortnightly until week 52 then monthly onwards.
- Side-effects checklist at the same time as physical health checks, including sedation, hypersalivation, constipation and any signs of immunosuppression or cardiac strain.

### Data collection

This project was approved by the Oxford Health NHS Foundation Trust Audit Management and Tracking service, with further ethical approval not required. All data on clozapine initiations that had occurred within the four teams during the study period were obtained from the Denzapine Monitoring System, because the Trust solely initiates clozapine under the brand name Denzapine. For each patient initiated on clozapine, a review of electronic health records was performed to ascertain the location of clozapine initiation. Additional information gathered included demographics, central non-re-challenge database check date, date of initiation and primary diagnosis. Patients with no prior exposure to clozapine were classified as first-time titrations; those who had been titrated previously were classified as re-titrations. For patients seen by TUNE-UP, a range of clinical measures were also obtained at baseline and, where possible, at discharge (listed in Box 2).

In order to quantify the duration of in-patient clozapine titrations, a prior service evaluation reviewed clinical notes for all patients initiating clozapine within Warneford Hospital (Oxford, UK) between July 2018 and December 2020. The average cost of in-patient bed-day was provided by the Trust.

### Statistical analysis

Counts of initiations were analysed using generalised linear models, with a log-link for observed exposure time to estimate initiation rates (implemented using the statsmodels package 0.14.4 in Python 3.12.8 on macOS).^26^ The primary analysis compared community initiations in the TUNE-UP year (period 3) with those in the other periods. For each outcome a Poisson regression was fitted, with the log of the period length as an offset. Over-dispersion was assessed using Pearson *χ* ^2^/degrees of freedom (with threshold set at 1.5; if greater than this, the negative binomial model was used). Incidence rate ratios (IRRs) with 95% confidence intervals and *P*-values are reported. A sensitivity analysis was also performed in which the post TUNE-UP period was excluded.

Secondary analyses involved three outcome groups with two or three analyses per group, and these were adjusted for multiple comparisons across the eight secondary tests using Benjamini–Hochberg (false discovery rate (FDR)) correction:

- Group 1: overall, i.e. first-time titrations and re-titrations, analysed together;
  - combined (i.e. community and in-patients);
  - in-patients only.
- Group 2: first-time titrations analysed separately;
  - community titrations only;
  - combined;
  - in-patients only.
- Group 3: re-titrations analysed separately;
  - community titrations only;
  - combined;
  - in-patients only.

An exploratory analysis was conducted for the clinical outcomes data, where paired changes were analysed using the Wilcoxon signed-rank test (two-sided). Baseline and discharge medians are reported, along with bootstrap 95% confidence intervals and two-sided *P*-values.

Statistical code and output tables are provided in the supplementary material available at https://doi.org/10.1192/bjo.2026.10988.

## Results

**Fig. 1:**
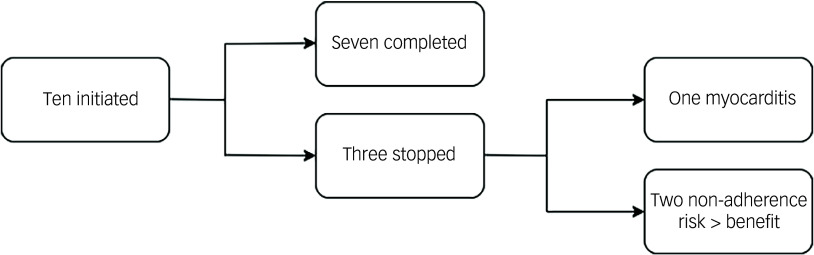
Flowchart of Treating Unmet Needs in Psychiatry (TUNE-UP) clinic initiations.

Fifty-four patients included in the four AMHTs of interest were recorded on the Denzapine Monitoring System as having potentially commenced clozapine treatment between September 2021 and January 2025. Three of these cases were registered but not actually titrated (due to these patients having relocated to the area). Of the remaining 51 cases, 29 were identified as first-time titrations and 22 as re-titrations. Demographic details for all patients receiving clozapine initiation are reported in Table 1, and referral reasons for TUNE-UP patients are reported in Table 2 (Fig. 1). The raw monthly breakdown of titration location is available in Supplementary Table 1).

**Table 1:** Demographic details of patients initiated on clozapine

Variables	Period 1 (12 months)	Period 2 (12 months)	Period 3 (Treating Unmet Needs in Psychiatry (TUNE-UP), 12 months)	Period 4 (4 months)
*N*	12	12	22	5
Median age, years (s.d.)	48 (13)	40 (18)	39 (16)	46 (10)
Male gender, *n* (%)	9 (75)	7 (58)	15 (68)	3 (60)
Ethnicity, *n* (%)				
White	10 (83)	7 (59)	17 (77)	3 (60)
Black or Black British	0 (0)	0 (0)	2 (9)	0 (0)
Asian or Asian British	2 (17)	4 (33)	2 (9)	1 (20)
Mixed	0 (0)	1 (8)	1 (5)	0 (0)
Not known	0 (0)	0 (0)	0 (0)	1 (20)
Diagnosis, *n* (%)				
Schizophrenia	8 (67)	6 (50)	15 (68)	2 (40)
Schizoaffective	3 (25)	6 (50)	4 (18)	3 (60)
Other	1 (8)	0 (0)	3 (14)	0 (0)
Location, *n* (%)				
In-patient	10 (83)	10 (83)	11 (50)	5 (100)
Community	2 (17)	2 (17)	11 (50)	0 (0)
Of which TUNE-UP	0 (0)	0 (0)	10 (45)	0 (0)
Treatment status, *n* (%)				
First-time titration, community | in-patient	5 (42)	6 (50)	15 (68)	3 (60)
	1 | 4	1 | 5	8 | 7	0 | 3
Re-titration, community | in-patient	7 (58)	6 (50)	7 (32)	2 (40)
	1 | 6	1 | 5	3 | 4	0 | 2

**Table 2:** Treating Unmet Needs in Psychiatry (TUNE-UP) referrals: demographics and referral reasons

Variables	All referrals	Clozapine initiations
*N*	47	10
Age, years (s.d.)	43.6 (13.9)	45.1 (15.8)
Male gender, *n* (%)	35 (74)	9 (90)
Referral reason, *n* (%)		
Positive symptoms	24 (51)	6 (60)
Negative symptoms	1 (2)	0 (0)
Cognitive symptoms	3 (6)	0 (0)
Mixed symptoms	7 (15)	1 (10)
Medication review	12 (26)	3 (30)

Ten patients were initiated by TUNE-UP, seven of whom were titrated onto a regular dose. Three patients had their titration discontinued – one of these was due to a recurrence of clozapine-related myocarditis, and in two patients non-adherence rendered the risk of continuing with titration as being greater than the potential benefits from ongoing treatment.

Of the ten patients titrated on clozapine by TUNE-UP, six had symptoms scored at discharge. In exploratory analyses there was a statistically significant improvement observed for scores in total PANSS and the Subjective Scale to Investigate Cognition in Schizophrenia (see Supplementary Table 2).

### Primary outcome

**Fig. 2:**
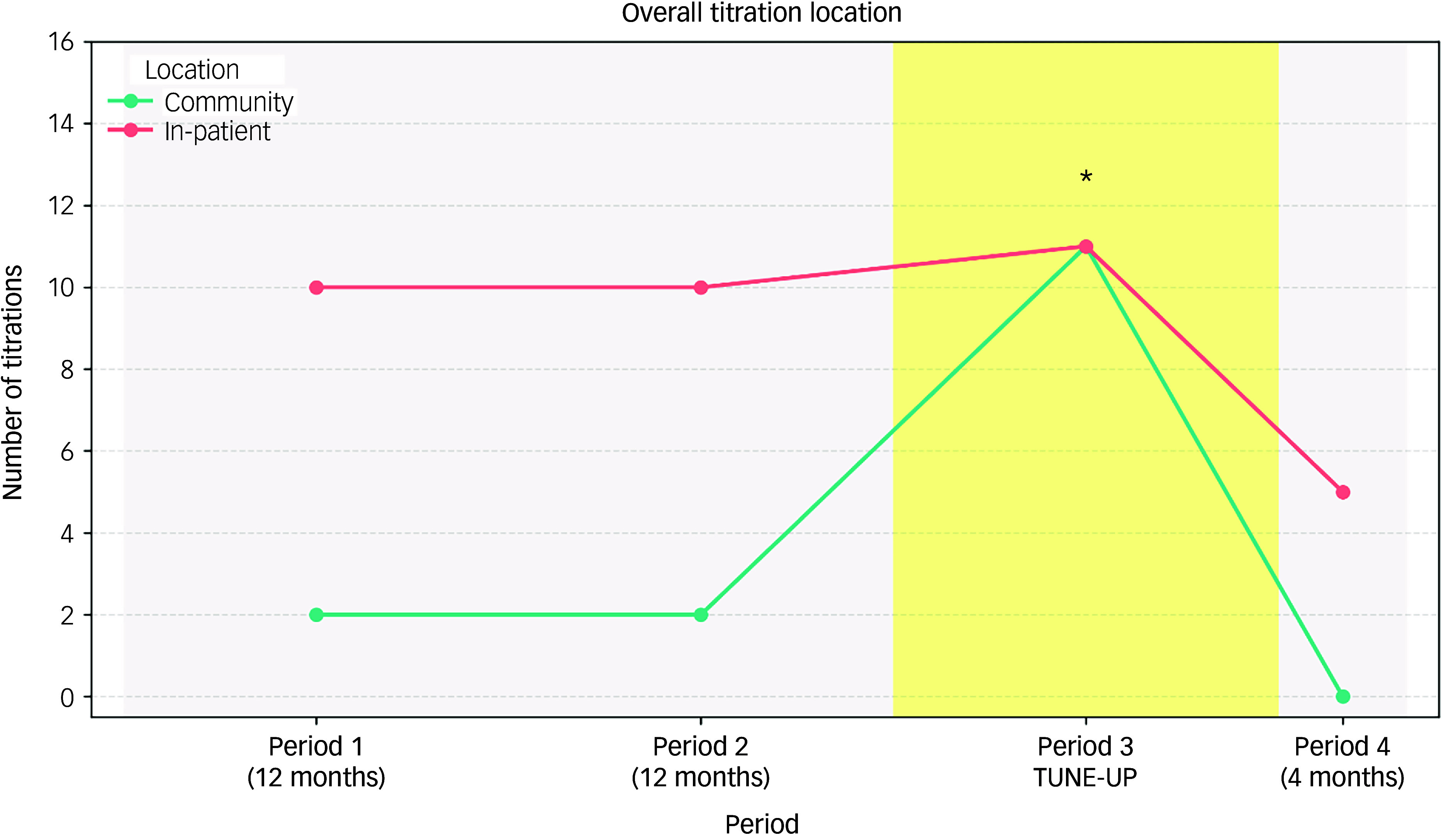
Treating Unmet Needs in Psychiatry (TUNE-UP) period is associated with significant increase in community clozapine initiations. *Statistically significant increase in community clozapine initiation during the TUNE-UP period *P* = 0.0015.

In the primary analysis there were significantly greater numbers of community clozapine initiations during the TUNE-UP period (11.0 per year) compared with the period when the service was not available (1.71 per year) (IRR = 6.42, 95% CI 2.04–20.2, *P* = 0.0015; see Fig. 2). In the sensitivity analysis, the increase in community clozapine initiations during the TUNE-UP period remained statistically significant when not including the post-TUNE-UP period (IRR = 5.5, 95% CI 1.75–17.3, *P* = 0.0035).

### Secondary outcomes

Within the secondary analyses (eight tests across three outcome groups), two further comparisons remained significant following FDR correction. There were significantly greater numbers of combined first-time titrations during the TUNE-UP period (15.0 per year) compared with the period when the service was unavailable (6.0 per year) (IRR = 2.5, 95% CI 1.21–5.18, FDR *q* = 0.041). There were also significantly greater numbers of community first-time titrations in the TUNE-UP period (8.0 per year) compared with when the service was unavailable (0.87 per year) when analysed separately (IRR = 9.33, 95% CI 1.98–44.0, FDR *q* = 0.021). There was a greater number of overall clozapine titrations (IRR = 1.77, 95% CI 1.02–3.08, *P* = 0.04, FDR *q* = 0.1), although this did not survive FDR correction. Complete model outputs are reported in Supplementary Table 3.

The service evaluation of in-patient clozapine duration identified 13 initiations, and these had a mean duration of 146 days (range 21–250 days). As of November 2025, the average cost for an acute in-patient bed stood at £764.31 per day.

## Discussion

Our findings show that a targeted out-patient service for community clozapine initiation is associated with a significant increase in community initiation rates. This increased rate of initiation was found primarily in patients who had not previously been titrated on clozapine (i.e. first-time titrations). There was no significant change in the rate of in-patient clozapine initiations in the period during which the service was running. Although previous work has demonstrated the cost-effectiveness of community clozapine services,^12^ to our knowledge this is the first demonstration that specialist community initiation clinics are associated with an increase in the frequency of community initiation.

The impact of the service was primarily associated with increasing community rates, rather than reducing in-patient admission rates. Many in-patients that are initiated are significantly unwell and require admission and, as such, this cohort would not be suitable for community initiation. When the service was unavailable, it appeared that most patients were offered clozapine initiation during periods of acute deterioration and in-patient admission. Therefore, the patients most likely to benefit from services such as TUNE-UP may be those with symptoms that impair functioning and who do not require in-patient admission. With restrictions of resources contributing to a lack of in-patient beds, and limited capacity of out-patient teams to initiate clozapine, it is likely that this cohort would continue to be suboptimally treated in the community and either continue in this way or deteriorate significantly as to require admission. First-time titrations were at their highest in the year during which this service was running, indicating that clinicians were able to trial clozapine in a greater number of TRS patients. An out-patient clozapine titration service may give clinicians the ability to offer clozapine to lower-risk TRS patients who would not otherwise receive this treatment, unless they deteriorated to the point of needing in-patient admission.

A high proportion of patients discontinue treatment with clozapine, with one of the factors previously shown to contribute to this being patients’ feelings of a lack of agency regarding decisions related to their treatment and health.^27^ Shared decision-making and person-centred care lead to improved adherence and outcomes.^27,28^ Community titration could allow patients greater autonomy, which may have positive effects on overall outcomes. Further qualitative research may be helpful in exploring patients’ attitudes and feedback regarding such a service.

Given the persistent underutilisation of clozapine, national efforts to increase patient and clinician awareness may be indicated to boost the appropriate prescription of this medication. Clearer pharmacological records of previous antipsychotics trialled, including dose, duration and monitoring of adherence (e.g. using measurement of antipsychotic levels), may help clinicians identify TRS as soon as criteria are fulfilled.^29^ Replicating the findings of this study in other trusts would contribute to their robustness.

Clinical guidelines advise offering clozapine to TRS patients at the earliest opportunity: a longer delay negatively impacts prognosis.^3,4^ The cost-effectiveness of clozapine treatment in TRS has been shown repeatedly, as have the benefits regarding life expectancy and suicidality.^30,31^ Despite this, clozapine is underutilised and delays in initiation average up to 5 years in the UK.^8,9,32,33^ There are significant differences in the rates of clozapine prescribing among different trusts, with one study from 2007 showing a persistent 5-fold difference in rates of clozapine prescription between 45 National Health Service (NHS) trusts.^34^ A more recent study in 2021 indicated that clozapine is grossly underutilised in the UK, with only a third of eligible patients receiving this treatment and a threefold variation in clozapine prescription in England.^35^ A previous study has shown a point prevalence of 56% of all patients under a general community mental health service meeting criteria for TRS, indicating a high rate of resistance in the community setting; half of these patients had never been trialled on clozapine.^36^ This reflects similar findings both in Europe and internationally.^37,38^

The underutilisation of clozapine results from a wide range of factors. Clinicians struggle to identify patients with TRS, and limitations of resources, and clinicians’ and patients’ attitudes towards clozapine, also play a significant role.^13,39^ Other barriers to timely treatment include practitioners’ anxiety regarding medical complications related to clozapine, their concerns regarding concordance, comorbid medical conditions and the logistics of initiation; and also patients’ concerns regarding tolerability and difficulty in adhering to blood test monitoring. Patients describe the necessity for hospital admission as the greatest barrier to agreeing to treatment with clozapine.^13^ Dedicated out-patient services focused on clozapine initiation may address some these barriers, and this has been identified by practitioners as a factor that would increase prescribing.^39^

There are also potential cost benefits. In the NHS trust of this study, the average cost per bed-day in an acute adult mental health ward in Oxfordshire was found to be £764.31, and in-patient admissions for clozapine titration typically last around 146 days. Based on this, the cost of the 10 additional titrations delivered by TUNE-UP would possibly have exceeded £1 000 000 if conducted in an in-patient setting. This figure, however, probably includes admissions due to symptomatic exacerbation rather than solely for the purpose of initiation. In a setting outside of Oxford Health, the cost of a 4-week, in-patient clozapine initiation has been estimated at £11 697, which would equate to £116 970 for 10 patients.^12^ It is important to note that these figures do not account for the potential longer-term savings associated with clozapine use, primarily due to reductions in future hospital admissions.^40^ A previous cost-effectiveness study of a similar service demonstrated the cost benefits of the approach.^12^ These data highlight the need for innovative, community-based approaches to increase clozapine initiation in a cost-effective manner.

### Strengths and limitations

The methodological strengths of this study include the ability to identify a comprehensive cohort of individuals commencing clozapine for the first time during the study period, and the ability to accurately identify their titration location from electronic records with minimal confounders of these data. The risk of selection bias of patients in this retrospective study was limited by using a centralised registry to identify all cases of clozapine initiation. In addition, the fact that the service was piloted for 12 months, and data were gathered following the termination of the pilot, provide a natural experiment which demonstrates that the findings are not reflective of more general long-term trends unrelated to the service.

Limitations of this study include the moderate total number of patients, the small cohort initiated by the TUNE-UP service and the lack of randomisation; furthermore, the observational nature is unable to ascribe causality to the effect of the intervention. A further possible limitation of this study is data incompleteness: in the case of re-titrations it is possible that, in cases where only a short break had occurred, the re-titration may not have been recorded on the Denzapine Monitoring Service database and would therefore have been missed in the analysis. This would not, however, affect the analysis of first-time titrations. The robustness of the clinical change measures is limited by the fact that they were obtained by unblinded clinicians and in only a small number of patients, and also by the potential for attrition bias. It is also possible that there may have been coincidental changes locally or nationally that could have influenced rates of community initiation and that might account for the observed results. This is unlikely, however, given the extremely low levels of clozapine prescribing seen both prior to the service being in operation and then subsequent to the service ceasing to offer clozapine initiation.

Potential weaknesses of the service model design include the need for patients to travel to the out-patient clinic for monitoring and clinical review, and the fragmentation of care between the parent team and TUNE-UP clinic. Strengths include intensive and focused clinical input for an often-neglected clinical population, whereby certain interventions may have a greater impact on health and functioning.

The findings of this study demonstrate that a targeted service that can titrate patients on clozapine in the community is associated with a significant increase in community clozapine initiation rates. When analysed separately, there was a significant increase in the rate of community and combined first-time titrations during the period during which this service was running. Patients titrated on clozapine through the service had improved symptom burden and functioning, although the sample size was small. There is now evidence showing that services for the community initiation of clozapine are associated with increased rates of community clozapine initiation, and these may provide clinical benefit to patient groups that are otherwise being treated suboptimally in the community.

## Supporting information

Ahmad Khan et al. supplementary material Ahmad Khan et al. supplementary material

## Data Availability

The data are available from the corresponding author on request.

## Supplementary material

The supplementary material is available online at https://doi.org/10.1192/bjo.2026.10988
