# Supplementary material for: Treating Unmet Needs in Psychiatry (TUNE-UP): targeted service increases out-patient initiations of clozapine
Source: BJPsych Open. 2026 Feb 26;12(2):e73. doi: 10.1192/bjo.2026.10988 (PMC12963841; doi:10.1192/bjo.2026.10988)
Supplement: Ahmad Khan et al. supplementary material [file S2056472426109880sup001.docx]

**TUNE-UP Paper Supplement**

| Year | Month | Total | Inpatient | Community | TUNE-UP | FTT IP | FTT C | RT IP | RT C |
| --- | --- | --- | --- | --- | --- | --- | --- | --- | --- |
| 1 | September | 1 | 1 | 0 | 0 | 1 | 0 | 0 | 0 |
| 1 | October | 3 | 3 | 0 | 0 | 0 | 0 | 3 | 0 |
| 1 | November | 0 | 0 | 0 | 0 | 0 | 0 | 0 | 0 |
| 1 | December | 0 | 0 | 0 | 0 | 0 | 0 | 0 | 0 |
| 1 | January | 1 | 1 | 0 | 0 | 0 | 0 | 1 | 0 |
| 1 | February | 0 | 0 | 0 | 0 | 0 | 0 | 0 | 0 |
| 1 | March | 2 | 2 | 0 | 0 | 1 | 0 | 1 | 0 |
| 1 | April | 3 | 2 | 1 | 0 | 1 | 0 | 1 | 1 |
| 1 | May | 1 | 1 | 0 | 0 | 1 | 0 | 0 | 0 |
| 1 | June | 0 | 0 | 0 | 0 | 0 | 0 | 0 | 0 |
| 1 | July | 0 | 0 | 0 | 0 | 0 | 0 | 0 | 0 |
| 1 | August | 1 | 0 | 1 | 0 | 0 | 1 | 0 | 0 |
| 2 | September | 1 | 1 | 0 | 0 | 1 | 0 | 0 | 0 |
| 2 | October | 3 | 3 | 0 | 0 | 0 | 0 | 3 | 0 |
| 2 | November | 0 | 0 | 0 | 0 | 0 | 0 | 0 | 0 |
| 2 | December | 0 | 0 | 0 | 0 | 0 | 0 | 0 | 0 |
| 2 | January | 1 | 1 | 0 | 0 | 1 | 0 | 0 | 0 |
| 2 | February | 0 | 0 | 0 | 0 | 0 | 0 | 0 | 0 |
| 2 | March | 2 | 2 | 0 | 0 | 1 | 0 | 1 | 0 |
| 2 | April | 3 | 2 | 1 | 0 | 1 | 0 | 1 | 1 |
| 2 | May | 1 | 1 | 0 | 0 | 1 | 0 | 0 | 0 |
| 2 | June | 0 | 0 | 0 | 0 | 0 | 0 | 0 | 0 |
| 2 | July | 0 | 0 | 0 | 0 | 0 | 0 | 0 | 0 |
| 2 | August | 1 | 0 | 1 | 0 | 0 | 1 | 0 | 0 |
| 3 | September | 0 | 0 | 0 | 0 | 0 | 0 | 0 | 0 |
| 3 | October | 6 | 2 | 4 | 3 | 1 | 4 | 1 | 0 |
| 3 | November | 1 | 1 | 0 | 0 | 1 | 0 | 0 | 0 |
| 3 | December | 2 | 2 | 0 | 0 | 2 | 0 | 0 | 0 |
| 3 | January | 5 | 3 | 2 | 2 | 2 | 2 | 1 | 0 |
| 3 | February | 1 | 1 | 0 | 0 | 0 | 0 | 1 | 0 |
| 3 | March | 3 | 1 | 2 | 2 | 1 | 1 | 0 | 1 |
| 3 | April | 1 | 0 | 1 | 1 | 0 | 0 | 0 | 1 |
| 3 | May | 0 | 0 | 0 | 0 | 0 | 0 | 0 | 0 |
| 3 | June | 2 | 1 | 1 | 1 | 0 | 0 | 1 | 1 |
| 3 | July | 1 | 0 | 1 | 1 | 0 | 1 | 0 | 0 |
| 3 | August | 0 | 0 | 0 | 0 | 0 | 0 | 0 | 0 |
| 4 | September | 0 | 0 | 0 | 0 | 0 | 0 | 0 | 0 |
| 4 | October | 4 | 4 | 0 | 0 | 3 | 0 | 1 | 0 |
| 4 | November | 1 | 1 | 0 | 0 | 0 | 0 | 1 | 0 |
| 4 | December | 0 | 0 | 0 | 0 | 0 | 0 | 0 | 0 |
| 4 | January | 0 | 0 | 0 | 0 | 0 | 0 | 0 | 0 |
|  |  |  |  |  |  |  |  |  |  |
|  | Total | 51 | 36 | 15 | 10 | 19 | 10 | 17 | 5 |

**SUPPLEMENT Table 1:** Monthly raw counts for initiations

| Outcome | N | Median Baseline (CI) | Median  Discharge (CI) | Wilcoxon p |
| --- | --- | --- | --- | --- |
| PANSS | 6 | 62.5 (58 – 81.5) | 46.5 (39 – 55) | 0.0312* |
| PANSS_pos | 6 | 26 (17.5 – 31.5) | 19 (11.5 – 22.5) | 0.0625 |
| BNSS | 5 | 26 (7 – 44) | 8 (3 – 14) | 0.0625 |
| SCIP | 6 | 60 (48 – 72) | 69.5 (49 – 73.5) | 0.1562 |
| SSTICS | 6 | 29 (27 – 36.5) | 22 (18 – 26) | 0.0312* |
| SOFAS | 6 | 50 (40 – 55) | 61.5 (42.5 – 67.5) | 0.0625 |

**SUPPLEMENT Table 2:** Analysis of clinical outcomes in TUNE-UP clozapine titrations

PANSS: Positive and Negative Syndrome Scale; BNSS: Brief Negative Syndrome Scale; CDS: Calgary Depression Scale; SCI: Sleep Condition Indicator; SSTICS: Subjective Scale to Investigate Cognition in Schizophrenia; SCIP: Screen for Cognitive Impairment in Psychiatry; SOFAS: Social and Occupational Functioning Assessment Scale; SDS: Sheehan Disability Scale.

^a^Complete discharge scales obtained for 6 participants (only 5 for BNSS). For CDS, SCI, and SDS discharge scores only obtained for two participants and so not analysed.

| Outcome | IRR (CI) | p_value | FDR q-value |
| --- | --- | --- | --- |
| Primary: Community (full) | 6.42 (2.04 – 20.2) | 0.0015* | - |
| Community (exclude Period 4) | 5.50 (1.75 – 17.3) | 0.0035* | - |
| G1: Community+Inpatient (full) | 1.77 (1.02 – 3.08) | 0.0434 | 0.098 |
| G1: Inpatient (full) | 1.03 (0.51 – 2.09) | 0.942 | 0.942 |
| G2 (FTT): Community (full) | 9.33 (1.98 – 44.0) | 0.0047 | 0.021* |
| G2 (FTT): Community+Inpatient (full) | 2.50 (1.21 – 5.18) | 0.0137 | 0.041* |
| G2 (FTT): Inpatient (full) | 1.36 (0.54 – 3.46) | 0.5168 | 0.723 |
| G3 (RT): Community (full) | 3.50 (0.58 – 20.9) | 0.17 | 0.306 |
| G3 (RT): Community+Inpatient (full) | 1.09 (0.44 – 2.67) | 0.8524 | 0.942 |
| G3 (RT): Inpatient (full) | 0.72 (0.23 – 2.20) | 0.5622 | 0.723 |

**SUPPLEMENT Table 3:** Initiation location results summary

In the period the TUNE-UP service was operational, there was a significant increase in the number of community clozapine initiations. This significance survived the sensitivity analysis where the post-TUNE-UP period was excluded. There were no changes to overall community and inpatient titrations when analysed together, or inpatient titrations when analysed separately. When first-time titrations were analysed separately, there was a significant increase in the rates of initiation in both community and combined (community and inpatient) titrations.

Code

Analysis

"""

harmonised_analysis.py

Single script that:

 - Runs Poisson GLM with log(exposure) offset for primary & secondary outcomes

 - Computes Quasi-Poisson (SE scaled) and Negative Binomial (if available)

 - Falls back to an aggregated rate-ratio (Wald) for small-sample cases

 - Produces one harmonised table (rows: outcome x model) with columns:

     outcome, model, dispersion, IRR, CI_lower, CI_upper, p_value

 - Selects preferred p-values for SECONDARY outcomes and applies Bonferroni/Holm/FDR

 - Saves results to CSVs

Requirements: numpy, pandas, statsmodels, scipy

pip install numpy pandas statsmodels scipy

"""

import numpy as np

import pandas as pd

import statsmodels.api as sm

from math import sqrt

from scipy.stats import norm

from statsmodels.stats.multitest import multipletests

# ------------------------------

# 1) INPUT DATA - edit here as needed

# ------------------------------

# DF1 - Primary Outcome + Secondary Outcome Group 1

df = pd.DataFrame({

    "period": [1,2,3,4],

    "year_label": ['Year 1','Year 2','Year 3','Year 4 (Observed 4 months)'],

    "community": [2,2,11,0],

    "inpatient": [10,10,11,5],   # your updated Year4 inpatient = 5 (observed 4 months)

    "exposure_months": [12,12,12,4]

})

df['tuneup'] = (df['period'] == 3).astype(int)

df['combined'] = df['community'] + df['inpatient']

# DF2 - Secondary Outcome Group 2 (FTT)

df2 = pd.DataFrame({

    "period": [1,2,3,4],

    "year_label": ['Year 1','Year 2','Year 3','Year 4 (Observed 4 months)'],

    "community": [1,1,8,0],

    "inpatient": [4,5,7,3],   # your updated Year4 inpatient = 3 (observed 4 months)

    "exposure_months": [12,12,12,4]

})

df2['tuneup'] = (df2['period'] == 3).astype(int)

df2['combined'] = df2['community'] + df2['inpatient']

# DF3 - Secondary Outcome Group 3 (RT)

df3 = pd.DataFrame({

    "period": [1,2,3,4],

    "year_label": ['Year 1','Year 2','Year 3','Year 4 (Observed 4 months)'],

    "community": [1,1,3,0],

    "inpatient": [6,5,4,2],   # your updated Year4 inpatient = 2 (observed 4 months)

    "exposure_months": [12,12,12,4]

})

df3['tuneup'] = (df3['period'] == 3).astype(int)

df3['combined'] = df3['community'] + df3['inpatient']

# ------------------------------

# 2) Helper functions

# ------------------------------

def fit_models_standardised(counts, exposures, tuneup, outcome_name):

    """

    Returns (DataFrame rows, meta)

    DataFrame has rows for Poisson, Quasi-Poisson, Negative Binomial.

    If Poisson cannot be fit, returns (None, {'error': ...})

    """

    rows = []

    X = sm.add_constant(tuneup)

    offset = np.log(exposures)

    y = counts.astype(float)

    # Try Poisson GLM

    try:

        pois = sm.GLM(y, X, family=sm.families.Poisson(), offset=offset).fit()

    except Exception as e:

        return None, {"error": str(e)}

    # Dispersion

    pearson_chi2 = (pois.resid_pearson**2).sum()

    dispersion = pearson_chi2 / pois.df_resid if pois.df_resid > 0 else np.nan

    # Poisson row

    try:

        irr_p = float(np.exp(pois.params['tuneup']))

        ci_low_p, ci_high_p = np.exp(pois.conf_int().loc['tuneup']).tolist()

        p_p = float(pois.pvalues['tuneup'])

    except Exception:

        irr_p, ci_low_p, ci_high_p, p_p = (np.nan, np.nan, np.nan, np.nan)

    rows.append({

        "outcome": outcome_name,

        "model": "Poisson",

        "dispersion": dispersion,

        "IRR": irr_p,

        "CI_lower": ci_low_p,

        "CI_upper": ci_high_p,

        "p_value": p_p

    })

    # Quasi-Poisson (scale SE by sqrt(dispersion))

    try:

        bse = pois.bse

        se_q = bse * sqrt(dispersion) if (not np.isnan(dispersion) and dispersion > 0) else bse

        coef = pois.params['tuneup']

        z_q = coef / se_q['tuneup']

        p_q = float(2*(1 - norm.cdf(abs(z_q))))

        ci_q_low = float(np.exp(coef - 1.96 * se_q['tuneup']))

        ci_q_high = float(np.exp(coef + 1.96 * se_q['tuneup']))

        irr_q = float(np.exp(coef))

    except Exception:

        irr_q, ci_q_low, ci_q_high, p_q = (np.nan, np.nan, np.nan, np.nan)

    rows.append({

        "outcome": outcome_name,

        "model": "Quasi-Poisson",

        "dispersion": dispersion,

        "IRR": irr_q,

        "CI_lower": ci_q_low,

        "CI_upper": ci_q_high,

        "p_value": p_q

    })

    # Negative Binomial

    try:

        nb = sm.GLM(y, X, family=sm.families.NegativeBinomial(), offset=offset).fit()

        irr_nb = float(np.exp(nb.params['tuneup']))

        ci_nb_low, ci_nb_high = np.exp(nb.conf_int().loc['tuneup']).tolist()

        p_nb = float(nb.pvalues['tuneup'])

        rows.append({

            "outcome": outcome_name,

            "model": "Negative Binomial",

            "dispersion": dispersion,

            "IRR": irr_nb,

            "CI_lower": ci_nb_low,

            "CI_upper": ci_nb_high,

            "p_value": p_nb

        })

    except Exception:

        rows.append({

            "outcome": outcome_name,

            "model": "Negative Binomial",

            "dispersion": dispersion,

            "IRR": np.nan,

            "CI_lower": np.nan,

            "CI_upper": np.nan,

            "p_value": np.nan

        })

    return pd.DataFrame(rows), {"poisson_obj": pois}

def aggregated_rate_ratio(counts, exposures, tuneup, outcome_name):

    """

    Aggregated rate-ratio (Wald approx on log(IRR)) used as fallback for small-sample.

    """

    tune_mask = (tuneup == 1)

    events_t = int(counts.loc[tune_mask].sum())

    expo_t = float(exposures.loc[tune_mask].sum())

    events_o = int(counts.loc[~tune_mask].sum())

    expo_o = float(exposures.loc[~tune_mask].sum())

    if events_t == 0 or events_o == 0 or expo_t == 0 or expo_o == 0:

        return pd.DataFrame([{

            "outcome": outcome_name,

            "model": "Aggregated",

            "dispersion": np.nan,

            "IRR": np.nan,

            "CI_lower": np.nan,

            "CI_upper": np.nan,

            "p_value": np.nan

        }])

    rate_t = events_t / expo_t

    rate_o = events_o / expo_o

    irr = rate_t / rate_o

    se_log = sqrt(1.0/events_t + 1.0/events_o)

    z = np.log(irr) / se_log

    p = float(2*(1 - norm.cdf(abs(z))))

    ci_low = float(np.exp(np.log(irr) - 1.96 * se_log))

    ci_high = float(np.exp(np.log(irr) + 1.96 * se_log))

    return pd.DataFrame([{

        "outcome": outcome_name,

        "model": "Aggregated",

        "dispersion": np.nan,

        "IRR": float(irr),

        "CI_lower": ci_low,

        "CI_upper": ci_high,

        "p_value": p

    }])

# ------------------------------

# 3) Run analyses (primary + secondaries)

# ------------------------------

primary_name = "Primary: Community (full)"

primary_df, _ = fit_models_standardised(df['community'], df['exposure_months'], df['tuneup'], primary_name)

if primary_df is None:

    primary_df = aggregated_rate_ratio(df['community'], df['exposure_months'], df['tuneup'], primary_name)

# Secondary 1a: community excluding Year 4

sec1a_name = "Secondary: Community (exclude Y4)"

df_no_post = df[df['period'] != 4].copy()

sec1a_df, _ = fit_models_standardised(df_no_post['community'], df_no_post['exposure_months'], df_no_post['tuneup'], sec1a_name)

if sec1a_df is None:

    sec1a_df = aggregated_rate_ratio(df_no_post['community'], df_no_post['exposure_months'], df_no_post['tuneup'], sec1a_name)

# Secondary 1b: combined full

sec1b_name = "Secondary: Community+Inpatient (full)"

sec1b_df, _ = fit_models_standardised(df['combined'], df['exposure_months'], df['tuneup'], sec1b_name)

if sec1b_df is None:

    sec1b_df = aggregated_rate_ratio(df['combined'], df['exposure_months'], df['tuneup'], sec1b_name)

# Secondary 1c: inpatient full

sec1c_name = "Secondary: Inpatient (full)"

sec1c_df, _ = fit_models_standardised(df['inpatient'], df['exposure_months'], df['tuneup'], sec1c_name)

if sec1c_df is None:

    sec1c_df = aggregated_rate_ratio(df['inpatient'], df['exposure_months'], df['tuneup'], sec1c_name)

# -------------------------

# Secondary Group 2 (FTT) - FULL only (no sensitivity)

sec2a_name = "G2 (FTT): Community (full)"

sec2a_df, _ = fit_models_standardised(df2['community'], df2['exposure_months'], df2['tuneup'], sec2a_name)

if sec2a_df is None:

    sec2a_df = aggregated_rate_ratio(df2['community'], df2['exposure_months'], df2['tuneup'], sec2a_name)

sec2b_name = "G2 (FTT): Community+Inpatient (full)"

sec2b_df, _ = fit_models_standardised(df2['combined'], df2['exposure_months'], df2['tuneup'], sec2b_name)

if sec2b_df is None:

    sec2b_df = aggregated_rate_ratio(df2['combined'], df2['exposure_months'], df2['tuneup'], sec2b_name)

sec2c_name = "G2 (FTT): Inpatient (full)"

sec2c_df, _ = fit_models_standardised(df2['inpatient'], df2['exposure_months'], df2['tuneup'], sec2c_name)

if sec2c_df is None:

    sec2c_df = aggregated_rate_ratio(df2['inpatient'], df2['exposure_months'], df2['tuneup'], sec2c_name)

# -------------------------

# Secondary Group 3 (RT) - FULL only (no sensitivity)

sec3a_name = "G3 (RT): Community (full)"

sec3a_df, _ = fit_models_standardised(df3['community'], df3['exposure_months'], df3['tuneup'], sec3a_name)

if sec3a_df is None:

    sec3a_df = aggregated_rate_ratio(df3['community'], df3['exposure_months'], df3['tuneup'], sec3a_name)

sec3b_name = "G3 (RT): Community+Inpatient (full)"

sec3b_df, _ = fit_models_standardised(df3['combined'], df3['exposure_months'], df3['tuneup'], sec3b_name)

if sec3b_df is None:

    sec3b_df = aggregated_rate_ratio(df3['combined'], df3['exposure_months'], df3['tuneup'], sec3b_name)

sec3c_name = "G3 (RT): Inpatient (full)"

sec3c_df, _ = fit_models_standardised(df3['inpatient'], df3['exposure_months'], df3['tuneup'], sec3c_name)

if sec3c_df is None:

    sec3c_df = aggregated_rate_ratio(df3['inpatient'], df3['exposure_months'], df3['tuneup'], sec3c_name)

# Stack into one harmonised table

full_results_table = pd.concat([

    primary_df,

    sec1a_df, sec1b_df, sec1c_df,

    sec2a_df, sec2b_df, sec2c_df,

    sec3a_df, sec3b_df, sec3c_df

], ignore_index=True)

# Ensure numeric columns are numeric and round for display

numcols = ['dispersion', 'IRR', 'CI_lower', 'CI_upper', 'p_value']

for c in numcols:

    if c in full_results_table:

        full_results_table[c] = pd.to_numeric(full_results_table[c], errors='coerce')

display_table = full_results_table.copy()

display_table[numcols] = display_table[numcols].round(4)

# ------------------------------

# 4) Multiple-testing correction for SECONDARY outcomes

# ------------------------------

# Build the list of all secondary outcome names (three from each group)

secondary_names = [

    sec1a_name, sec1b_name, sec1c_name,   # Group 1 - sensitivity + combined + inpatient

    sec2a_name, sec2b_name, sec2c_name,   # Group 2 - FTT (full only)

    sec3a_name, sec3b_name, sec3c_name    # Group 3 - RT (full only)

]

preferred_list = []

for out in secondary_names:

    sub = full_results_table[full_results_table['outcome'] == out]

    if sub.empty:

        preferred_list.append((out, np.nan, None))

        continue

    disp = sub['dispersion'].iloc[0]

    nb_row = sub[sub['model'] == 'Negative Binomial']

    if (not nb_row.empty) and (not np.isnan(nb_row['p_value'].values[0])) and (not np.isnan(disp)) and (disp > 1.5):

        pref_p = float(nb_row['p_value'].values[0]); pref_model = 'Negative Binomial'

    else:

        pois_row = sub[sub['model'] == 'Poisson']

        if (not pois_row.empty) and (not np.isnan(pois_row['p_value'].values[0])):

            pref_p = float(pois_row['p_value'].values[0]); pref_model = 'Poisson'

        else:

            agg_row = sub[sub['model'] == 'Aggregated']

            if (not agg_row.empty) and (not np.isnan(agg_row['p_value'].values[0])):

                pref_p = float(agg_row['p_value'].values[0]); pref_model = 'Aggregated'

            else:

                pref_p = np.nan; pref_model = None

    preferred_list.append((out, pref_p, pref_model))

labels = [p[0] for p in preferred_list]

raw_pvals = np.array([p[1] for p in preferred_list], dtype=float)

raw_pvals_for_adj = np.where(np.isnan(raw_pvals), 1.0, raw_pvals)

# Apply three corrections

bonf = multipletests(raw_pvals_for_adj, alpha=0.05, method='bonferroni')

holm = multipletests(raw_pvals_for_adj, alpha=0.05, method='holm')

fdr = multipletests(raw_pvals_for_adj, alpha=0.05, method='fdr_bh')

correction_df = pd.DataFrame({

    'outcome': labels,

    'preferred_model': [p[2] for p in preferred_list],

    'raw_p': raw_pvals,

    'bonf_p': bonf[1], 'bonf_signif': bonf[0],

    'holm_p': holm[1], 'holm_signif': holm[0],

    'fdr_p': fdr[1], 'fdr_signif': fdr[0]

})

# ------------------------------

# 5) Output: print + save CSVs

# ------------------------------

print("\n=== Harmonised results table (one row per outcome x model) ===\n")

print(display_table.to_string(index=False))

print("\n=== Preferred p-values for SECONDARY outcomes and multiple-testing corrections ===\n")

print(correction_df.to_string(index=False))

display_table.to_csv("harmonised_results_table.csv", index=False)

correction_df.to_csv("secondary_pvalue_corrections.csv", index=False)

# Also make a single-row preferred summary (one chosen estimate per outcome)

def pick_preferred_row(out):

    sub = full_results_table[full_results_table['outcome'] == out]

    if sub.empty:

        return {"outcome": out, "model": None, "dispersion": np.nan, "IRR": np.nan, "CI_lower": np.nan, "CI_upper": np.nan, "p_value": np.nan}

    disp = sub['dispersion'].iloc[0]

    # prefer NB if overdispersion

    if (not np.isnan(disp)) and (disp > 1.5):

        nb = sub[sub['model'] == 'Negative Binomial']

        if (not nb.empty) and (not np.isnan(nb['p_value'].values[0])):

            r = nb.iloc[0]

            return {"outcome": out, "model": "Negative Binomial", "dispersion": r['dispersion'], "IRR": r['IRR'], "CI_lower": r['CI_lower'], "CI_upper": r['CI_upper'], "p_value": r['p_value']}

    # else prefer Poisson

    pois = sub[sub['model'] == 'Poisson']

    if (not pois.empty):

        r = pois.iloc[0]

        return {"outcome": out, "model": "Poisson", "dispersion": r['dispersion'], "IRR": r['IRR'], "CI_lower": r['CI_lower'], "CI_upper": r['CI_upper'], "p_value": r['p_value']}

    # fallback to aggregated

    agg = sub[sub['model'] == 'Aggregated']

    if (not agg.empty):

        r = agg.iloc[0]

        return {"outcome": out, "model": "Aggregated", "dispersion": r['dispersion'], "IRR": r['IRR'], "CI_lower": r['CI_lower'], "CI_upper": r['CI_upper'], "p_value": r['p_value']}

    # ultimate fallback

    return {"outcome": out, "model": None, "dispersion": np.nan, "IRR": np.nan, "CI_lower": np.nan, "CI_upper": np.nan, "p_value": np.nan}

# Build the preferred summary safely and round only existing columns

all_outcomes_for_summary = [primary_name] + secondary_names  # includes all 9 secondaries

summary_rows = [pick_preferred_row(o) for o in all_outcomes_for_summary]

preferred_summary = pd.DataFrame(summary_rows)

# Round only columns that exist

for c in ['dispersion', 'IRR', 'CI_lower', 'CI_upper', 'p_value']:

    if c in preferred_summary.columns:

        preferred_summary[c] = pd.to_numeric(preferred_summary[c], errors='coerce').round(4)

print("\n=== Preferred single-estimate summary (per outcome) ===\n")

print(preferred_summary.to_string(index=False))

preferred_summary.to_csv("preferred_single-estimate_summary.csv", index=False)

print("\nSaved CSVs: 'harmonised_results_table.csv', 'secondary_pvalue_corrections.csv', 'preferred_estimates_summary.csv'")

# End script

Clinical Outcomes

import numpy as np

import pandas as pd

from scipy.stats import wilcoxon

from math import sqrt

# -------------------------

# Input data (your arrays)

# -------------------------

data_dict = {

    "PANSS":       (np.array([57, 59, 61, 64, 75, 88]), np.array([38, 48, 58, 40, 45, 52])),

    "PANSS_pos":   (np.array([13, 25, 22, 29, 27, 34]), np.array([8, 20, 25, 18, 15, 20])),

    "BNSS":        (np.array([7, 31, 10, 44, 26]),       np.array([3, 6, 9, 14, 8])),

    "SCIP":        (np.array([53, 60, 60, 43, 71, 73]),  np.array([58, 69, 70, 40, 70, 77])),

    "SSTICS":      (np.array([28, 30, 36, 28, 37, 26]),  np.array([26, 22, 26, 20, 16, 22])),

    "SOFAS":       (np.array([50, 40, 50, 50, 60, 40]),  np.array([70, 40, 60, 63, 65, 45])),

}

# -------------------------

# Helper: percentile bootstrap CI

# -------------------------

def bootstrap_ci(data, func, n_boot=5000, alpha=0.05, random_state=12345):

    """

    Percentile bootstrap CI for func(data). Returns (lo, hi).

    data: 1-D numpy array

    func: function(data) -> scalar (e.g. np.median)

    """

    rng = np.random.default_rng(random_state)

    n = len(data)

    boots = np.empty(n_boot)

    for i in range(n_boot):

        sample = rng.choice(data, size=n, replace=True)

        boots[i] = func(sample)

    lo = np.percentile(boots, 100 * (alpha/2))

    hi = np.percentile(boots, 100 * (1 - alpha/2))

    return lo, hi

# -------------------------

# Loop: compute medians+CI and Wilcoxon p

# -------------------------

rows = []

for name, (baseline, discharge) in data_dict.items():

    if len(baseline) != len(discharge):

        raise ValueError(f"{name}: baseline/discharge different lengths")

    n = len(baseline)

    # median and bootstrap CI for baseline

    try:

        med_b = float(np.median(baseline))

        med_b_lo, med_b_hi = bootstrap_ci(baseline, np.median, n_boot=5000, random_state=12345)

    except Exception:

        med_b = np.nan

        med_b_lo, med_b_hi = (np.nan, np.nan)

    # median and bootstrap CI for discharge

    try:

        med_d = float(np.median(discharge))

        med_d_lo, med_d_hi = bootstrap_ci(discharge, np.median, n_boot=5000, random_state=12345)

    except Exception:

        med_d = np.nan

        med_d_lo, med_d_hi = (np.nan, np.nan)

    # Wilcoxon signed-rank p-value for paired differences (baseline - discharge)

    try:

        _, w_p = wilcoxon(baseline, discharge, zero_method='wilcox', correction=False)

    except Exception:

        w_p = np.nan

    rows.append({

        "Outcome": name,

        "N": n,

        "Median_baseline": round(med_b, 3) if med_b == med_b else np.nan,

        "Median_baseline_CI_lo": round(med_b_lo, 3) if med_b_lo == med_b_lo else np.nan,

        "Median_baseline_CI_hi": round(med_b_hi, 3) if med_b_hi == med_b_hi else np.nan,

        "Median_discharge": round(med_d, 3) if med_d == med_d else np.nan,

        "Median_discharge_CI_lo": round(med_d_lo, 3) if med_d_lo == med_d_lo else np.nan,

        "Median_discharge_CI_hi": round(med_d_hi, 3) if med_d_hi == med_d_hi else np.nan,

        "Wilcoxon_p": round(w_p, 4) if w_p == w_p else np.nan

    })

results_df = pd.DataFrame(rows)

# order columns exactly as requested

cols = ["Outcome", "N",

        "Median_baseline", "Median_baseline_CI_lo", "Median_baseline_CI_hi",

        "Median_discharge", "Median_discharge_CI_lo", "Median_discharge_CI_hi",

        "Wilcoxon_p"]

results_df = results_df[cols]

# print and save CSV

pd.set_option('display.max_columns', None)

print("\nClinical outcomes (median, 95% CI, Wilcoxon p):\n")

print(results_df.to_string(index=False))

results_df.to_csv("clinical_outcomes_medians_wilcoxon.csv", index=False)

print("\nSaved: clinical_outcomes_medians_wilcoxon.csv")
